# A kernel density estimation-based approach for quantifying O-GlcNAcylation dysregulation in cancer from gene expression data

**DOI:** 10.1093/bioadv/vbag045

**Published:** 2026-02-13

**Authors:** Rastko Stojšin, Jinlian Wang, Hongfang Liu

**Affiliations:** Center for Translational AI Excellence and Applications in Medicine, D. Bradley McWilliams School of Biomedical Informatics, The University of Texas Health Science Center at Houston, Houston, TX 77030, United States; Center for Translational AI Excellence and Applications in Medicine, D. Bradley McWilliams School of Biomedical Informatics, The University of Texas Health Science Center at Houston, Houston, TX 77030, United States; Center for Translational AI Excellence and Applications in Medicine, D. Bradley McWilliams School of Biomedical Informatics, The University of Texas Health Science Center at Houston, Houston, TX 77030, United States

## Abstract

**Motivation:**

O-GlcNAcylation, a dynamic post-translational modification regulated by O-GlcNAc transferase (OGT) and O-GlcNAcase (OGA), influences critical biological processes and is dysregulated in cancers. Direct measurement of O-GlcNAcylation dysregulation is challenging due to its instability and low-throughput nature, limiting large-scale studies. However, the regulatory simplicity of this system and the availability of transcriptomic data enable inference of dysregulation from OGT and OGA expression.

**Results:**

We introduce a nonparametric kernel density estimation-based approach to quantify O-GlcNAcylation dysregulation using joint OGT and OGA expression. In simulated datasets with varied expression patterns and controlled dysregulation levels, our method consistently outperformed canonical metrics in quantifying dysregulation. In TCGA data from six cancer types, inferred regulation scores were significantly lower in cancer samples (0.25–0.30 vs. 0.49–0.51) and showed strong distributional differences (Kolmogorov–Smirnov *P* values <5.95e-11; D-statistics >0.31) compared to those from healthy samples. The scores also allow for accurate classification of cancer status (AUROC: 0.71–0.75) and generalized well to external datasets without retraining. This transcriptomics-based framework offers a scalable approach for interpretable quantification of O-GlcNAcylation dysregulation in cancer.

**Availability and implementation:**

The code and datasets used in this study are freely available at https://github.com/wonder-ai/O-GlcNAcylation_Project under an open-source license.

## 1 Introduction

O-GlcNAcylation is a dynamic and reversible post-translational modification (PTM) involving of the attachment of N-acetylglucosamine (GlcNAc) to serine/threonine residues of proteins (Hart *et al.* 2011). This process is uniquely regulated by just two enzymes: O-GlcNAc transferase (OGT), which adds GlcNAc, and O-GlcNAcase (OGA), which removes it—together these genes maintain tight control over modification levels (Ong *et al.* 2018). This PTM regulates key signaling pathways (e.g. PI3K/AKT, MYC, NF-κB) and plays an essential role in fundamental cellular processes including transcription, metabolism, cell cycle progression, and stress response (Hart and Akimoto 2009, Bond and Hanover 2015, Wu *et al.* 2024). O-GlcNAcylation dysregulation is increasingly implicated in many diseases, most notably cancers, where it contributes to altered cellular metabolism, chromosomal instability, apoptosis resistance, and immune evasion—hallmarks that promote tumorigenesis, progression, metastasis, and therapy resistance (Ma and Vosseller 2014, Ferrer *et al.* 2016, Akella *et al.* 2019, Rodrigues *et al.* 2020, Wu *et al.* 2024).

Direct measurement of O-GlcNAcylation remains technically challenging in clinical and research settings due to its ubiquity, rapid cycling, and *ex vivo* instability (Thompson *et al.* 2018). Although current techniques—such as mass spectrometry, protein-specific immunodetection, and chemoenzymatic labeling—can be effective in controlled laboratory settings, they are highly sensitive to sample handling, require enrichment steps prone to bias, and remain low-throughput (Thompson *et al.* 2018). This limits the feasibility of the large-scale profiling needed to study the role of the dysregulation of this modification in cancer. Fortunately, the broad availability of transcriptomic data provides a practical alternative for inferring O-GlcNAcylation dysregulation in cancer.

Numerous computational methods have been developed to quantify biological dysregulation in cancer. These include information-theory based metrics (e.g. mutual information), correlation-based methods (e.g. canonical correlation, Pearson, Spearman), dimensionality reduction techniques (e.g. PCA), distance-based (Mahalanobis), distributional comparisons (Jensen-Shannon divergence), and statistical descriptors such as coefficient of variation (Alter *et al.* 2000, Shechtman 2013, Seoane *et al.* 2014, van Wieringen and van der Vaart 2015, Schissler *et al.* 2015, Fang *et al.* 2016, Wang *et al.* 2021, Huang *et al.* 2024, Ludwik *et al.* 2024). While useful in some contexts, these methods often struggle to capture the behavior of coordinated regulatory systems like OGT and OGA. Because they rely on marginal statistics, assume fixed functional forms, or aggregate across populations, existing methods often fail to model joint regulatory dynamics or yield interpretable, sample-specific results. These limitations hinder their application in high-resolution analyses and downstream integration (Berretta and Moscato 2010, Kreeger and Lauffenburger 2010).

To address these limitations, we introduce a kernel density estimation (KDE)-based framework for inferring O-GlcNAcylation dysregulation from the joint expression of OGT/OGA. Our approach is a flexible, non-parametric method that estimates joint probability distributions without assuming a specific functional form, making it well-suited for modeling linear, nonlinear, and unknown regulatory relationships (Izenman 1991, Stanisław 2018). O-GlcNAcylation’s single-writer/single-eraser regulation—together with its well-established dysregulation in cancer—provides a biologically grounded rationale for inference, where joint density estimation on healthy samples defines high-probability regions of intact regulation. KDE-based regulation scores—derived from the probability density of healthy samples—are interpretable and support both sample-level inference and cohort-level comparisons. These properties allow for easy integration with downstream modeling or visualization pipelines. While demonstrated here using OGT/OGA in cancer, this approach can generalize to other tightly regulated gene pairs and phenotypes where balanced expression is critical, and dysregulation is phenotype-relevant.

To evaluate the effectiveness of our KDE-based regulation score, we designed a multi-stage validation using simulated and clinical datasets. We benchmarked our KDE approach using simulated datasets with predefined levels of dysregulation, comparing it to a wide range of established metrics. To assess generalizability, we applied the method—without retraining—to six external datasets, evaluating consistency across varied cohorts. This evaluation strategy supports the method’s utility for scalable, transcriptomics-based inference.

## 2 Methods

### 2.1 Data acquisition and processing

We evaluated our KDE-based approach using three data sources: synthetic datasets for controlled benchmarking, TCGA expression data for clinical analysis, and GEO datasets for external validation. Extraction and processing steps for all datasets—including data acquisition, clinical filtering, and batch correction—are fully documented and publicly available in our GitHub repository (https://github.com/wonder-ai/O-GlcNAcylation_Project).

Synthetic Data: We simulated biologically plausible gene–gene expression scenarios across four regulatory shapes—linear, exponential, parabolic, and multicluster. For each shape, dysregulation was introduced by increasing noise in the “cancerous” group at three levels (1.0×, 1.5×, and 2.0× compared to healthy group), with 100 simulations per condition.

TCGA Data: Gene expression and clinical data were obtained for the six largest cancer types from TCGA: blood and bone marrow (*n = *7224), lung (*n = *1996), kidney (*n = *1842), breast (*n = *1594), thyroid (*n = *1402), and uterine (*n = *1168). All accessed via the Genomic Data Commons (Grossman *et al.* 2016, The Cancer Genome Atlas Research Network 2024).

GEO Data: Gene expression and clinical metadata were obtained for matching cancer types: blood and bone marrow (GSE13159, *n = *2096) (Kohlmann *et al.* 2008, Haferlach *et al.* 2010), lung (GSE19804, *n = *120) (Lu *et al.* 2010, 2015), kidney (GSE66271 and GSE53757, *n = *169) (von Roemeling *et al.* 2014, Liep *et al.* 2016, Wotschofsky *et al.* 2016), breast (GSE42568, *n = *121) (Clarke *et al.* 2013), thyroid (GSE58545, *n = *44) (Rusinek *et al.* 2015), and uterine (GSE17025, *n = *103) (Day *et al.* 2011, Day and McDade 2013).

### 2.2 Regulation score

We quantified OGT and OGA dysregulation using KDE, identifying regions of highest healthy tissue density. Regulation scores, derived from KDE-based density maps of OGT/OGA expression, were assigned to each sample. We calculated the KDE at each point using the following formula:


(1) $$\mathrm{KDE}(\mathrm{gene}1,\;\mathrm{gene}2)=\frac{1}{nh}\sum_{i=1}^{n}K(\frac{\mathrm{gene}1-{\mathrm{gene}1}_{i}}{h},\frac{\mathrm{gene}2-{\mathrm{gene}2}_{i}}{h})$$

where *K* is the kernel function, *h* is the bandwidth, gene1 and gene2 are the expression levels of the sample point being evaluated, ${\mathrm{gene}1}_{i}$ and ${\mathrm{gene}2}_{i}$ are the expression levels of each datapoint respectively, n is the number of healthy samples, and IQR is the interquartile range of the gene1 and gene2 expression levels used to determine the bandwidth h:


(2)  h=2×IQR×n-13

To ensure scale consistency and interpretability, we defined a KDE threshold (${\mathrm{KDE}}_{t})$ such that 50% of the healthy samples fall within the corresponding high-density region. This threshold does not represent a strict binary boundary between regulated and dysregulated states but instead it serves to normalize the distributions so that the average healthy sample has a regulation score of 0.5. Each sample is assigned a regulation score, proportional to the KDE value, using the following formula:


(3) Regulation scorei={KDEi-min⁡(KDE)KDEt-min⁡(KDE)×0.50.5+KDEi-KDEtmax⁡(KDE)-KDEt×0.5 if KDEi ≤ KDEtif KDEi > KDEt

where ${\mathrm{KDE}}_{i}$ is the KDE for each sample and ${\mathrm{KDE}}_{t}$ is the threshold at which half of the healthy samples fall inside and half outside. This produces a continuous scale ranging from 1 (lowest density, indicating strong dysregulation) to 0 (highest density, indicating high consistency with healthy expression). This allows for interpretable scoring and helps facilitate cross cohort comparisons.

### 2.3 Benchmarking on synthetic data

To assess the versatility of the KDE-based regulation scores, we generated synthetic datasets simulating joint expression of two genes under healthy and progressively more dysregulated cancer-like conditions. Four expression patterns—linear, exponential, parabolic, and multicluster—were modeled to represent a range of biologically plausible regulatory relationships. Dysregulation in the “cancerous” group was simulated by progressively increasing expression noise at three levels: no additional noise (1.0×), moderate noise (1.5×), and high noise (2.0×). Each condition was simulated 100×.

For metrics that lack sample-level resolution (e.g. mutual information, canonical correlation, PCA variance, correlation coefficients, coefficient of variation, Jensen-Shannon divergence), we summarized dysregulation as a healthy-to-cancer ratio or divergence (Alter *et al.* 2000, Berretta and Moscato 2010, Kreeger and Lauffenburger 2010, Liu *et al.* 2012, Shechtman 2013, Seoane *et al.* 2014, van Wieringen and van der Vaart 2015, Wang *et al.* 2015, Fang *et al.* 2016, Li *et al.* 2016, Lapek *et al.* 2017, Wang *et al.* 2020, 2021, Frost 2024, Huang *et al.* 2024, Ludwik *et al.* 2024). Metrics were considered robust if (i) at 1.0× noise they correctly inferred no dysregulation between healthy and cancer cohorts (1.0 for ratio-based metrics, 0.5 for divergence), and (ii) increased monotonically with noise, confirmed by one-sided Mann–Whitney U tests (*P < .*01) between successive noise levels.

We evaluated distance-based metrics that support sample-level scoring [e.g. Manhattan, Euclidean, Chebyshev, Cosine, and Mahalanobis (Schissler *et al.* 2015, Frost 2024)] by computing their classification performance using area under the receiver operating characteristic curve (AUROC). For each regulatory shape and noise condition, we generated independent training and testing datasets simulating healthy and cancerous joint gene expression. The KDE model was fit, and the distances metrics were calculated relative to healthy training sample then applied to the unseen test data. AUROC was computed to quantify the ability of each metric to distinguish cancerous from healthy samples. This procedure was repeated across 100 simulations per configuration to evaluate robustness and sensitivity to underlying dysregulation. Metrics were considered robust in this setting if AUROC distributions for the 1.0× condition centered near 0.5 (indicating no discriminative signal) and progressively increased with higher noise levels.

### 2.4 Clinical evaluation on TCGA data

To assess whether KDE-based regulation scores reflect clinically meaningful dysregulation, we applied the method to six cancer types from The Cancer Genome Atlas (TCGA). These datasets provide gene expression and clinical annotations across healthy and cancerous tissue, allowing evaluation in settings where dysregulation is known to occur but cannot be directly measured. Because no ground-truth quantitative dysregulation scores exist in clinical data, aggregate-level methods (e.g. mutual information, canonical correlation, PCA) cannot be meaningfully benchmarked. These approaches produce cohort-wide statistics that lack per-sample resolution and are therefore not directly comparable to our KDE-based score. We evaluated regulation scores based on three criteria: (i) whether cancer samples exhibit higher average dysregulation, reflected by regulation scores below the healthy reference value of 0.5; (ii) whether score distributions differ significantly between cancerous and healthy tissue; and (iii) whether KDE-based scores improve classification performance over expression-derived features and comparable distance metrics.

### 2.5 Kolmogorov-Smirnov testing

We performed batch correction using covariance-based joint distribution alignment. We used the two-sample Kolmogorov–Smirnov (KS) test to assess differences in regulation score distributions between healthy and cancer samples (Massey 1951). This non-parametric test compares the empirical cumulative distribution functions (CDFs) of two samples, making no assumptions about their underlying distributions. It is particularly well-suited for detecting both location shifts and shape differences, which may arise due to dysregulation. The KS statistic is defined as:


(4) $$\mathrm{KS}=\;\sup_{x}|F_{1}(x)-\;F_{2}(x)|$$

where $F_{1}(x)$ and $F_{2}(x)$ are the empirical CDFs of the regulation scores in the healthy and cancer groups, respectively. The supremum (sup) denotes the maximum absolute difference between the two CDFs across all *x*. For each cancer type, a *P* value was computed to assess whether the score distributions diverged significantly, with lower *P* values indicating stronger evidence of dysregulation in the cancer group.

### 2.6 Predictive modeling

To assess whether the KDE-based regulation score added predictive value beyond its component features, we implemented a five-fold cross-validation (CV) framework using logistic regression classifiers. In each fold, we computed KDE-based regulation scores using the healthy samples from the training set and applied them to both training and test sets. This ensured that the KDE reference distributions were constructed independently of the test data.

We compared models trained on the following feature sets: KDE-based regulation score, raw expression of OGT and OGA, their multiplicative interaction term (OGT × OGA), a derived ratio (OGT/OGA) and single-gene expression along with distance-based metrics. We evaluated model performance using AUROC, averaged across all folds. This design tests whether the KDE score captures complex joint regulatory patterns—such as non-linear dependencies or conditional co-expression—not trivially modeled by simple combinations of OGT and OGA. By benchmarking against these component-based baselines, we isolate the added discriminative power of the KDE-based score while maintaining interpretability grounded in its biological underpinnings.

### 2.7 External validation on GEO data

To evaluate the generalizability of the KDE-based regulation score, we applied the method to six external gene expression datasets from the Gene Expression Omnibus (GEO), each matched to a TCGA cancer type. These cohorts were selected to assess performance on independently collected datasets with different preprocessing pipelines and patient populations. We performed batch correction using covariance-based joint distribution alignment, an established method in cross-platform genomic studies, that preserves relationships between expressions while aligning across datasets (Lee *et al.* 2014, Somekh *et al.* 2019). This approach enabled the direct application of TCGA-trained KDE models to GEO datasets for regulation score generation—without retraining or modification. Classification performance of cancer status using these scores was compared against the same expression-derived and distance-based features assessed in the TCGA analysis to ensure consistency in benchmarking across datasets.

## 3 Results

### 3.1 Synthetic benchmarking

We conducted benchmarking of the KDE-based regulation scores using simulated datasets representing four biologically feasible regulatory relationships (linear, exponential, parabolic, and multicluster) and varying dysregulation levels (1.0×, 1.5×, 2.0×), with 100 simulations per setting.

**Figure 1:**
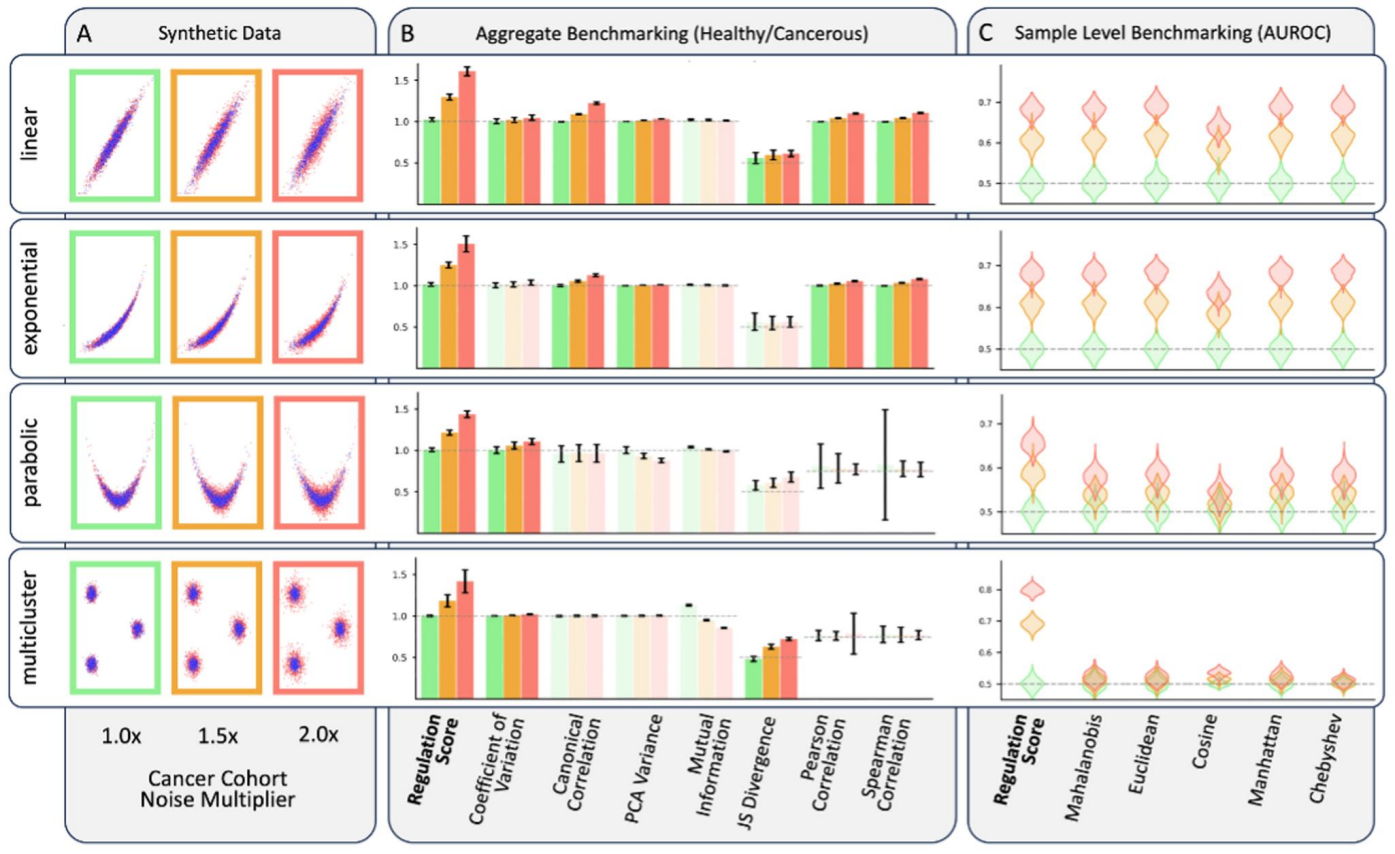
Synthetic benchmarking. (A) Synthetic expression data under four regulatory patterns and three noise levels. (B) Metric distributions with robust metrics highlighted. (C) AUROC distributions (100 simulations).

For cohort-level discrimination, only the KDE-based regulation score consistently demonstrated robust performance across all scenarios (Fig. 1). It consistently showed no spurious signal at baseline and monotonic increases with higher levels of simulated dysregulation accurately distinguishing dysregulated from well-regulated cohorts. In contrast, other metrics failed to maintain stable discrimination across regulatory patterns, often captured spurious signals, and/or had difficulty detecting dysregulation in nonlinear relationships.

At the sample level, the regulations scores supported the most accurate prediction of cohort status across all tested configurations (Fig. 1). Models trained on regulation scores then tested on newly generated data (with same parameters) achieved AUROC values near 0.5 in the absence of dysregulation—indicating no false positives—with consistent performance improvements as noise increased. Distance-based alternatives showed weaker and inconsistent separation, particularly struggling with nonlinear regulatory patterns where they failed to maintain predictive accuracy. These results establish the KDE-based approach as both accurate and robust, capable of detecting cohort and sample-level dysregulation in a wide range of nonlinear and heterogeneous expression patterns while avoiding false positives in well-regulated systems.

### 3.2 Clinical evaluation

We evaluated our KDE-based dysregulation inference and its clinical utility across six cancer types of TCGA. These datasets provide gene expression and clinical annotations for healthy and cancerous tissues, enabling assessment of O-GlcNAcylation dysregulation in physiologically relevant contexts. Regulation scores were evaluated using three criteria: (i) tumor samples should exhibit higher dysregulation; (ii) score distributions between healthy and cancerous tissue should be statistically distinguishable; and (iii) KDE-based scores should improve classification performance compared to expression-derived features alone and should outperform other distance metrics.

**Figure 2:**
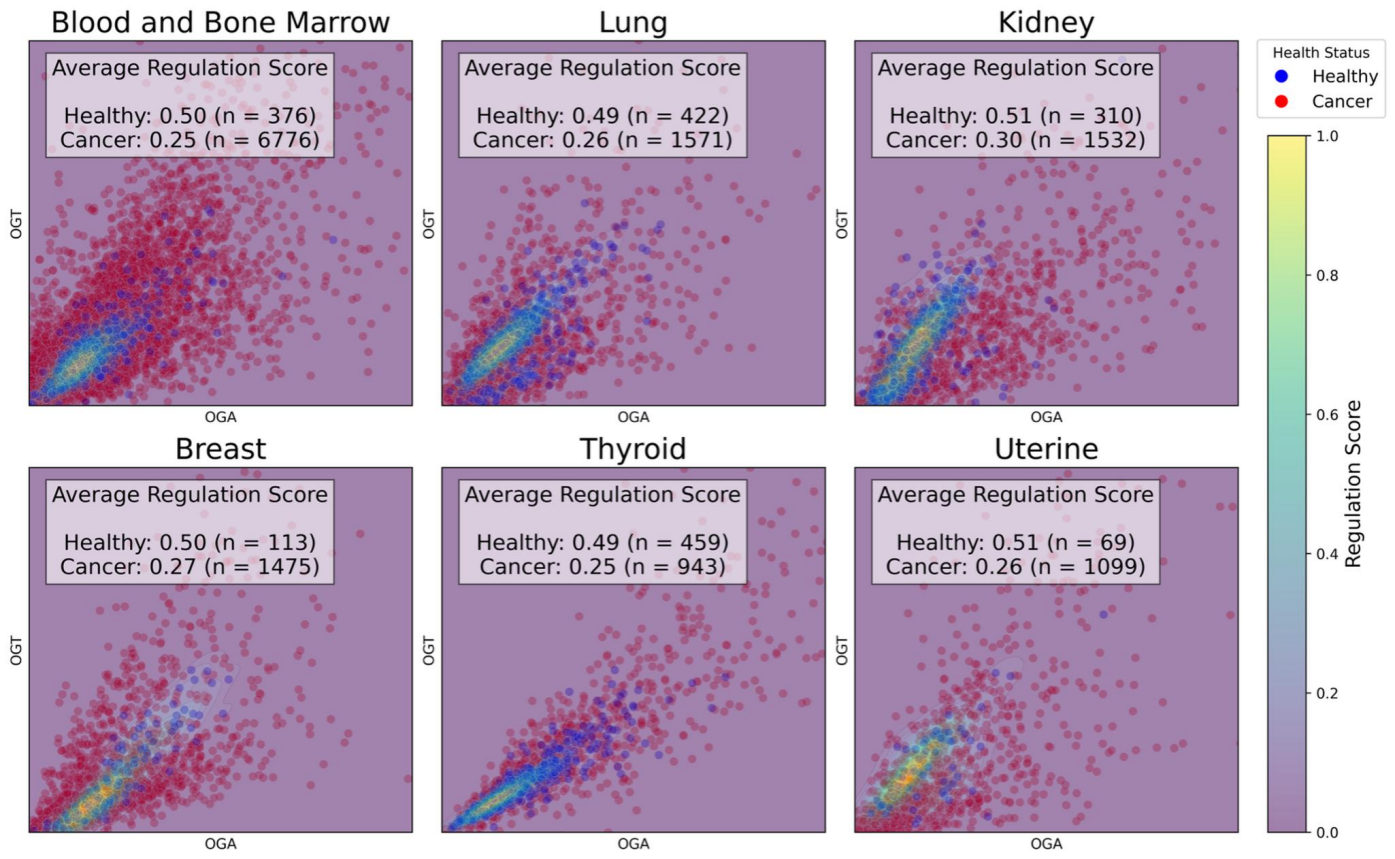
Regulation score distributions. Each panel includes cancerous and healthy samples, with assigned values shown via a color-coded heatmap for each evaluated cancer type.

OGT and OGA expression patterns, overlaid on the KDE landscape, revealed that healthy samples clustered within high-density regions, while cancerous samples were broadly dispersed (Fig. 2). Across cancers, average regulation scores were consistently lower in tumor samples: 2.0-fold lower in blood and bone marrow, thyroid, and uterine cancer; 1.9-fold in lung and breast; and 1.7-fold in kidney. These results suggest that O-GlcNAcylation dysregulation is common across tumor types and that the KDE-based score captures this divergence in a quantifiable, interpretable manner.

**Figure 3:**
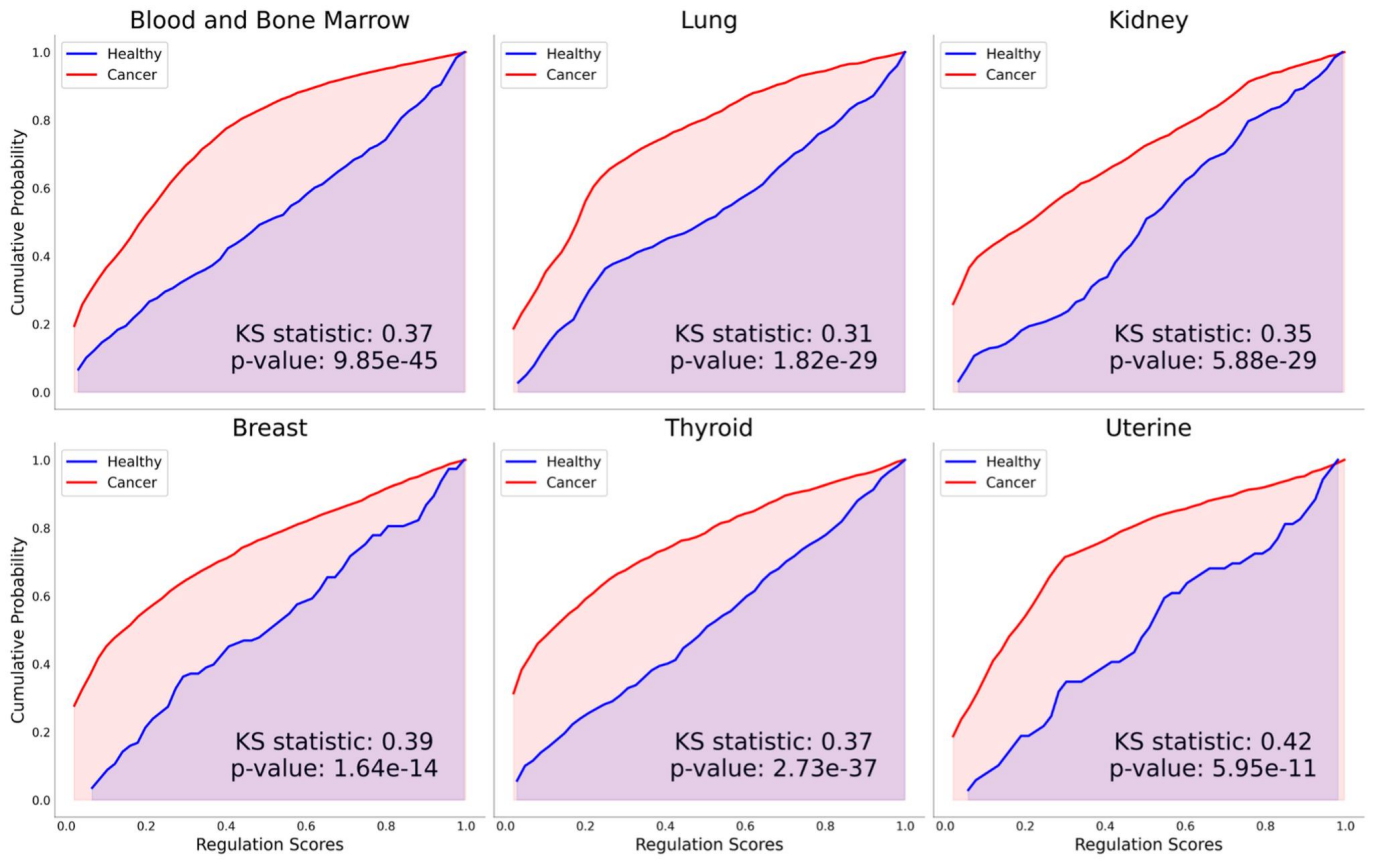
Cumulative distribution plots. Cumulative distribution plots of KDE-based O-GlcNAcylation regulation scores for six cancer types. Each panel shows distributions for healthy and cancerous samples, with KS statistics and P values displayed.

Two-sample KS tests revealed significant differences in regulation score distributions between healthy and cancer samples. Regulation scores in cancerous tissue were consistently shifted toward lower values relative to healthy controls, with all six cancer types exhibiting significant differences (*P* < 5.95 × 10^−11^) (Fig. 3). These results validate the sensitivity of the KDE-based score confirming its ability to distinguish disease-relevant changes in joint expression.

**Figure 4:**
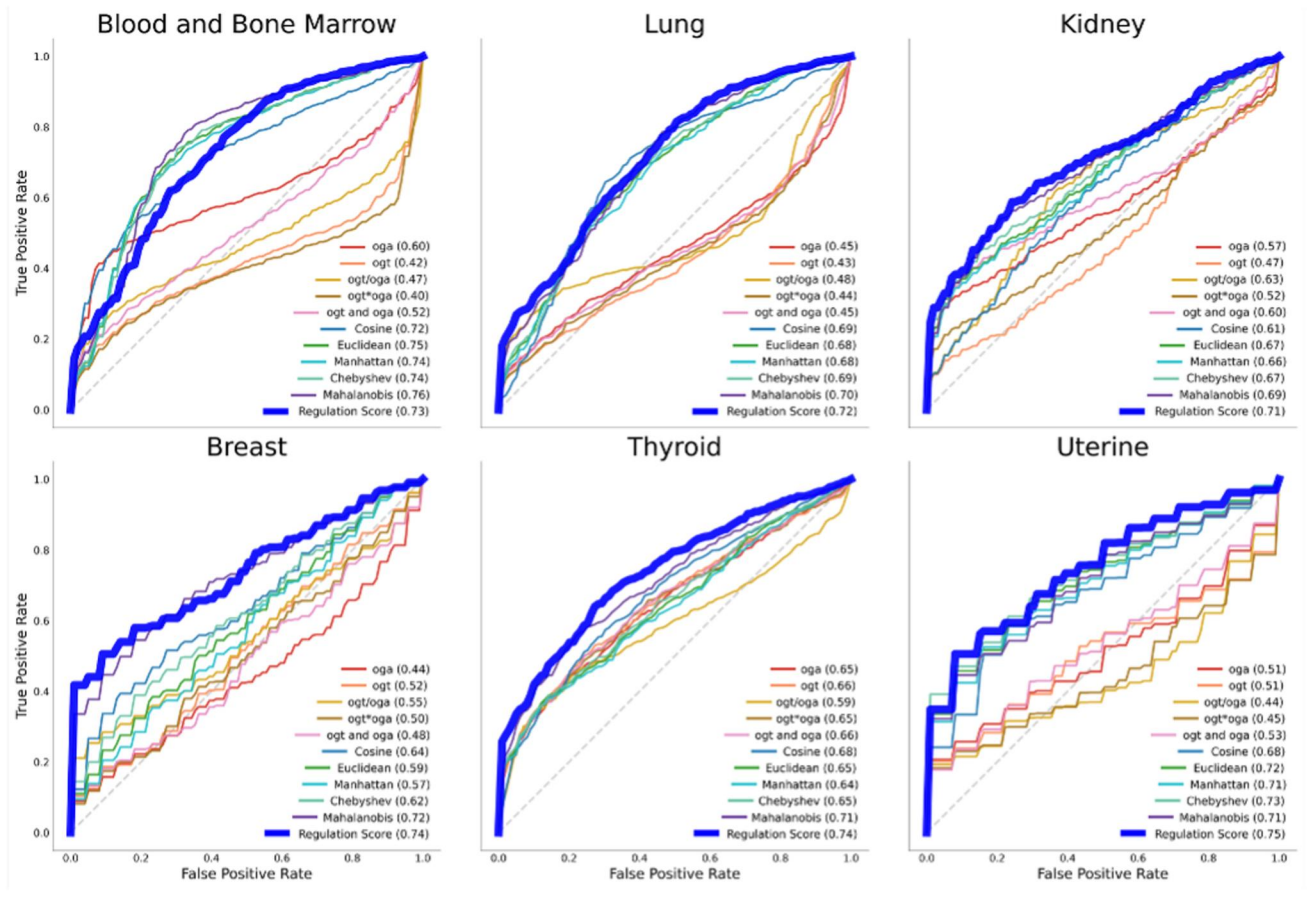
ROC curves. ROC curves comparing the performance of metrics in distinguishing cancerous from healthy samples for six cancer types.

Classifiers using KDE-based scores outperformed models based on other derived expression features and distance metrics in all but one of the cancer types evaluated (Fig. 4). The average AUROC across cancer types was highest for our approach (0.73). It outperformed other approaches for all but the blood and bone marrow dataset where our score (0.73) closely matched the best-performing metric (0.76). These findings demonstrate that the KDE-based approach captures joint regulatory patterns not reflected in simple linear or ratio-based combinations—providing high classification performance while retaining biological interpretability.

### 3.3 External validation

When applied out of the box to six independent GEO datasets, the KDE-based regulation scores consistently indicated higher dysregulation in cancer samples and successfully distinguished cancerous from healthy samples across all matched cancer types. Except for batch correction to align marginal expression distributions, we applied the method without any retraining or reparameterization.

**Figure 5:**
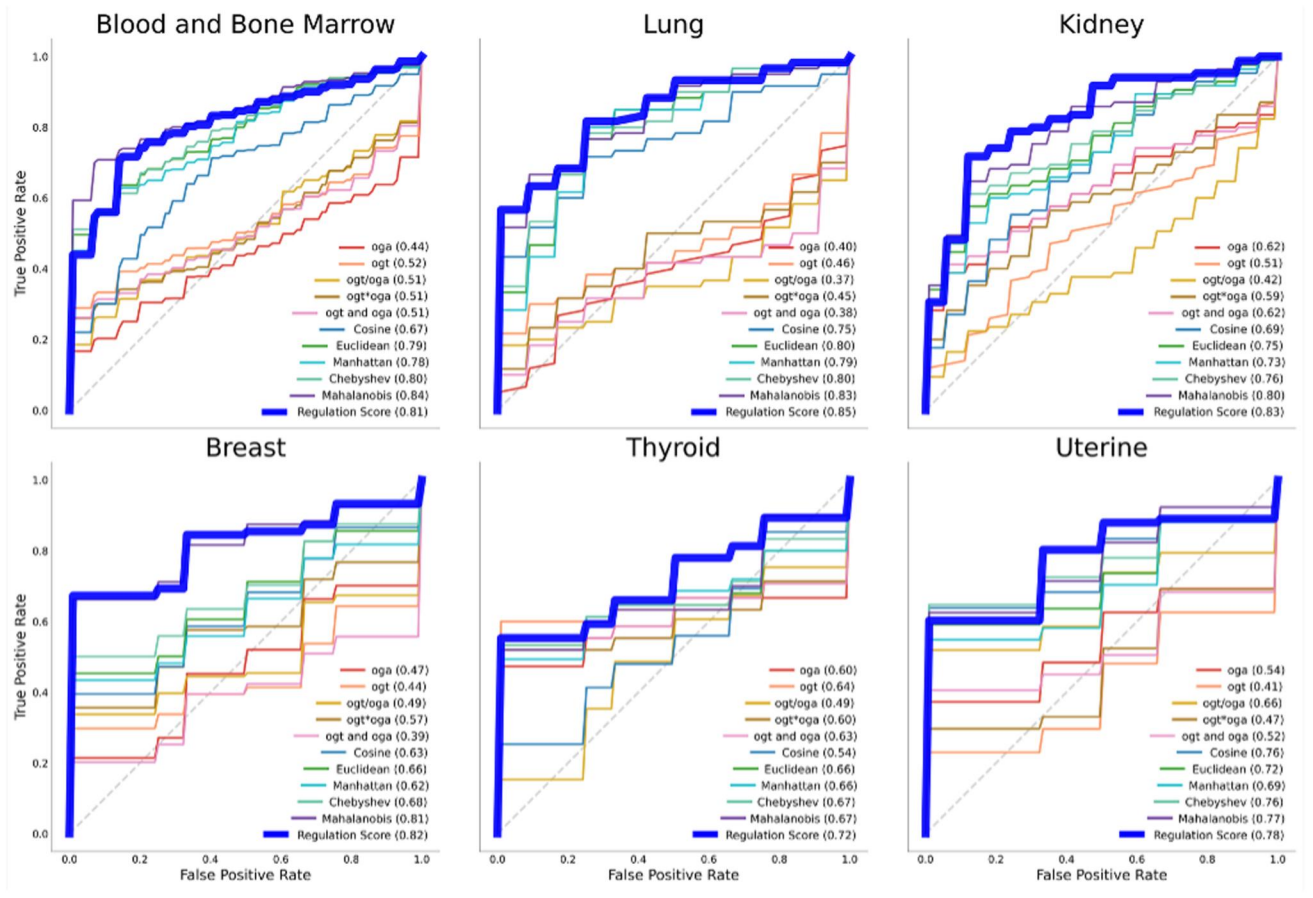
Validation ROC curves. ROC curves comparing the performance of metrics in distinguishing cancerous from healthy samples for external six cancer types.

Predictive performance closely matched TCGA benchmarks, surpassing all baseline methods despite platform, preprocessing, and cohort differences. The average AUROC was 0.80, outperforming all other approaches (Fig. 5). This consistent outperformance across cohorts indicates that the KDE-based approach captures biologically relevant regulatory structure that remains stable across experimental settings. The one exception was the blood and bone marrow dataset, where performance was comparable (0.81 vs. 0.84). The method’s ability to retain discriminative power without retraining supports its potential for scalable deployment in clinical and research contexts.

## 4 Discussion

In this study, we developed and validated a KDE-based regulation score to quantify dysregulation in O-GlcNAcylation from joint of OGT and OGA expression. Our approach consistently outperformed traditional statistical, correlation-based, and distance-based metrics in identifying dysregulated expression patterns across both synthetic and clinical datasets This result likely reflects the strong linear relationship between OGT/OGA expression and health status in this tissue. Unlike previous methods that rely on marginal statistics, linear assumptions, or population-level aggregation, our method offers interpretable, sample-specific scoring grounded in the biological regulation of a two-enzyme system. This probabilistic interpretation—based on empirical density rather than arbitrary cutoffs—provides intuitive insight into how far a given sample deviates from expected healthy expression. This interpretability may be particularly valuable in translational contexts, such as risk stratification or personalized monitoring.

Performance on synthetic data confirmed the flexibility of our approach across diverse regulatory scenarios, including linear, nonlinear, and multicluster relationships. Notably, the KDE-based score demonstrated robust detection of dysregulation under simulated noise, maintaining sensitivity and specificity, while alternative metrics failed to capture subtle nonlinear changes or produced false positives in well-regulated contexts. When applied to TCGA cancer datasets, KDE-based scores distinguished healthy from cancerous samples across all six evaluated cancer types, with consistent statistical significance (*P* < 5.95 × 10^−11^, KS test). Furthermore, classifiers built on these scores achieved the highest average AUROC (0.73), outperforming all distance metrics and expression-derived features. This trend held across most cancer types and was especially notable in nonlinear expression regimes where traditional methods underperformed. The one exception to this trend occurred in the blood and bone marrow dataset, where a distance-based metric narrowly outperformed the KDE-based score (AUROC 0.73 vs. 0.76). This result likely reflects the strong linear relationship between OGT/OGA expression and health status in this tissue (Pearson *R* = 0.80 and $R^{2}=$ 0.64). In such a context, distance metrics naturally excel. However, the KDE-based method still performed comparably, demonstrating its robustness even when linear assumptions favor competing techniques. In more complex expression landscapes—such as those observed in kidney, uterine, or breast cancer—the KDE method captured dysregulation more effectively.

Beyond performance, our method demonstrated strong generalizability: KDE scores trained on TCGA healthy data generalized without retraining to independent GEO datasets. Despite differences in platforms, sample composition, and preprocessing pipelines, the method retained discriminative power and ranked among the top-performing approaches, underscoring its potential for clinical and cross-cohort application.

In a clinical context, KDE-based scoring could aid in identifying dysregulated samples early in disease progression or in stratifying patients by degree of metabolic disruption. Although primarily intended for research applications, it may support retrospective or preclinical studies where healthy references are available, and future normalization strategies could enable broader translational use Moreover, integration with proteomic or phospho-proteomic data could link transcript-level dysregulation to downstream functional consequences, enhancing biological insight and enabling multi-omic extensions of the model. O-GlcNAcylation regulates key signaling networks such as PI3K/AKT, NF-κB, and MYC, which are frequently altered in cancer. The consistent dysregulation detected by our score may represent downstream effects within these pathways, which will be examined through pathway enrichment analyses in future work. While promising, this approach has some limitations. First, the use of OGT and OGA transcript levels as proxies for O-GlcNAcylation assumes that mRNA abundance reflects functional enzyme activity. Although these genes are the only known writer and eraser of O-GlcNAc modifications, additional regulatory factors—such as enzyme kinetics, protein stability, and substrate availability—may influence modification levels. Secondly, relying on healthy reference samples may introduce batch effects or sample imbalance, which could impact score calibration. Nonetheless, we addressed this via joint distribution alignment during external validation and by designing the regulation score to be relative to the modeled healthy density, rather than absolute expression levels. A related concern is the use of healthy tissue samples to define the reference baseline for regulation. This introduces a potential circularity: disease status is assessed based on deviation from a healthy-derived model. However, this design is biologically justified. It establishes a normative regulatory framework against which pathological deviations can be meaningfully interpreted, much like z-scores or reference intervals in clinical diagnostics. Finally, our method does not account for molecular subtypes or intratumoral heterogeneity, which may be important for prognosis and therapy selection. Future extensions could address this by incorporating stratified or hierarchical KDE models.

In summary, the KDE-based framework offers a biologically grounded, statistically robust, and interpretable framework for quantifying O-GlcNAcylation dysregulation. It performs competitively across a range of data shapes, maintains stability across cohorts, and provides insight into gene-level regulatory perturbations with sample-level resolution. These results not only validate its use for O-GlcNAcylation, but also suggest broader applicability to other tightly regulated gene systems in health and disease. Future studies may extend this work by incorporating dynamic transcriptomic profiles, exploring regulation beyond OGT/OGA, and integrating proteomic measurements to connect transcript-level dysregulation with functional downstream effects.

## Data Availability

The data and code underlying this article is available in our GitHub, at https://github.com/wonder-ai/O-GlcNAcylation_Project
